# Combined Fluorescent-Chromogenic *In Situ* Hybridization for Identification and Laser Microdissection of Interphase Chromosomes

**DOI:** 10.1371/journal.pone.0060238

**Published:** 2013-04-02

**Authors:** Nerea Paz, Amaia Zabala, Félix Royo, África García-Orad, José L. Zugaza, Luis A. Parada

**Affiliations:** 1 Department of Genetics, Faculty of Medicine and Dentistry, University of the Basque Country, Leioa, Spain; 2 CIC BioGUNE-CIBERehd, Derio, Spain; 3 Department of Genetics, Physical Anthropology and Animal Physiology, Faculty of Science and Technology, University of the Basque Country, Leioa, Spain; 4 Achucarro Basque Center for Neuroscience, Zamudio, Spain; 5 Institute of Experimental Pathology, UNSa-CONICET, Salta, Argentina; 6 IKERBASQUE, Basque Foundation for Science, Bilbao, Spain; The Chinese University of Hong Kong, Hong Kong

## Abstract

Chromosome territories constitute the most conspicuous feature of nuclear architecture, and they exhibit non-random distribution patterns in the interphase nucleus. We observed that in cell nuclei from humans with Down Syndrome two chromosomes 21 frequently localize proximal to one another and distant from the third chromosome. To systematically investigate whether the proximally positioned chromosomes were always the same in all cells, we developed an approach consisting of sequential FISH and CISH combined with laser-microdissection of chromosomes from the interphase nucleus and followed by subsequent chromosome identification by microsatellite allele genotyping. This approach identified proximally positioned chromosomes from cultured cells, and the analysis showed that the identity of the chromosomes proximally positioned varies. However, the data suggest that there may be a tendency of the same chromosomes to be positioned close to each other in the interphase nucleus of trisomic cells. The protocol described here represents a powerful new method for genome analysis.

## Introduction

Eukaryotic genomes are highly organized within the cell nucleus. Several studies have demonstrated the relevance of such organization for biological processes [1]. Chromosome territories (CT) constitute the most conspicuous feature of nuclear architecture and they exhibit non-random distribution patterns in the interphase nucleus [2], [3]. It is generally accepted that the nucleus core is occupied by gene-dense chromosomes or active regions of the genome, whereas the nuclear periphery, typically rich in heterochromatin, contains gene-poor chromosomes and less active domains [4]. Apart from being associated with silent chromosomal domains, the nuclear periphery has also been related to genome stabilization [5].

Fluorescent *in situ* hybridization (FISH) has emerged as an indispensable tool for studying the spatial organization of the genome with high precision. Labeled probes targeting whole chromosomes or chromosomal regions allow their direct visualization, both in metaphase and interphase [6]. Such painting probes can be generated from DNA isolated by metaphase-chromosome microdissection, followed by amplification and labeling with modified nucleotides by degenerate oligonucleotide primer-PCR (DOP-PCR). The procedure includes a step of universal amplification, which is particularly efficient at amplifying single copies of chromosomes for the production of paints or for performing other cytogenetic applications where only small amounts of DNA are available, such as from single cells or small pieces of microdissected tissue [7]. The chromogenic *in situ* hybridization (CISH) technique, on the other hand, is a suitable alternative to FISH. CISH produces a permanent chromosome stain by using peroxidase- or alkaline phosphatase-labeled reporter antibodies that interact with the hybridized DNA probe, which are subsequently detected using an enzymatic reaction [8]. The main advantage of CISH over FISH is that it can be viewed with a bright-field microscope.

In an effort to identify the chromosomes 21 that are proximally positioned in the interphase nucleus, we developed a novel combination strategy that exploits the advantages of both FISH and CISH. Our method involves cell preparation on special slides, hybridization with chromosome painting probes, and detection with a fluorescent-labeled antibody followed by colorimetric detection of this same antibody. Then, the processed interphase nucleus is subjected to chromosome laser-microdissection, and microsatellite allele genotyping is performed for chromosome identification. We have applied this approach to distinguish the identity of homologous chromosomes 21 that localize in close proximity in cells from humans with DS.

## Materials and Methods

### Cell Culture and Chromosome Preparation

The human lymphocyte cells lines GM03714 and 4710-176, derived from a cytogenetically normal individual and a patient with DS due to an extra copy of chromosome 21, respectively, were kindly donated by Prof. S. Antonarakis (University of Geneva, Switzerland) [9]. These authors obtained the informed consent for human samples, and their study was approved by the ethics committee of the Geneva University Hospital [9]. The cell lines were cultured in RPMI-Glutamax media containing 10% fetal bovine serum and 1% penicillin/streptomycin (Gibco Life Technologies, USA) at 37°C in a 5% CO_2_ atmosphere. Chromosome preparation was performed according to standard protocols [10]. Briefly, colcemid (0.04 µg/ml) was added to the media two hours prior harvesting and cells were collected by centrifugation at 800 rpm for 10 min. The cells were rinsed successively in buffer PBS 0.3X without Ca/Mg, KCl 0.056 M. Finally, the cells were fixed with a 3∶1 methanol:acetic acid solution and stored at −20°C. The approval for the study was provided by the Ethical Committee of the Basque Country Department of Health, and was performed according to the Declaration of Helsinki Principles.

### Cell Preparations

Cell spreading was done on special slides covered with a Poly Ethylene Naphthalate (PEN) membrane (Laser P.A.L.M., Germany), which permits the isolation of the material during the laser microdissection procedure. The slides (membranes) were irradiated with UV (254 nm) for 30 min, dehydrated in ethanol 100%, dipped into water, air-dried and then stored at −20°C until use. For analysis, cell preparations were dropped directly onto the PEN membrane surface and dried overnight at 37°C. Chromosome positioning analysis was performed on cells fixed following protocols designed for the preservation of the three dimensional structure of the nuclei previously described [10].

### FISH-probe for Chromosome 21

DNA from HSA21 was purchased from Cambio (Cambridge, UK) and labeled with digoxigenin-11dUTP (Roche Biochemicals, Switzerland) using DOP-PCR according previously described protocols [6]. The FISH probe was prepared by mixing 5 µL of digoxigenin-labeled HSA21 DNA (200 ng) with 4 µL of human COT1 DNA (4 µg; Invitrogen, USA) and 4 µL of salmon sperm DNA (40 µg; Invitrogen, USA). Then, the DNA was precipitated by adding 0.1 parts/volume of 3 M sodium acetate (pH 5.2) and 3 parts/volume of 100% ethanol, and collected by centrifugation (12000 rpm, 30 min, 4°C). The DNA pellet was resuspended in 12 µL hybridization buffer (10% dextran sulphate and 50% formamide in 2X saline-sodium citrate buffer (SSC), pH 7.0).

### Hybridization and Signal Detection

For each slide, a 12 µL aliquot of HSA21-probe was applied directly onto the membrane. The slide was covered with a 20×60 mm cover slip and sealed with rubber cement. Then, a pre-hybridization step was performed by incubating the preparation at 37°C for one hour. Target chromosomes and DNA-probe were denatured together in a thermal plate at 80°C for 8 min and immediately transferred to a moist chamber for incubation at 37°C during 72 hours. After the hybridization, the cover slips were carefully removed to avoid damaging the membrane. The slides were dipped in a series of washing solutions at 45°C (50%Formamide/2X SSC, 1XSSC, and 4XSSC/Tween20 0.1% (4T)) for 10 min in each of them. A final 5 min wash was carried out in the 4T solution at room temperature. The preparations were incubated with a blocking solution consisting of albumin 3% in 4T (4A) for 30 min at 37°C.

The chromosome 21 signal was initially detected by an antibody against digoxigenin conjugated with fluorescein (FITC; Roche Biochemicals, Switzerland). A 1∶200 dilution of the antibody (in buffer 4A) was applied to the preparation, and incubated for 60 min at 37°C in a humidified chamber protected from light. After a 5 min wash with buffer 4T, the slides were visualized under a fluorescence microscope to verify appropriate hybridization. If so, CISH was performed subsequently using the DuoCISH kit (Dako, Glostrup, Denmark), according the manufacturer instructions. Briefly, the slides were dipped in washing buffer 1XTBS/Tween20 0.05% (TBST) twice for 5 min each time, and incubated with a peroxidase blocking solution containing hydrogen peroxide 3%/15 mM sodium azide for 5 minutes at room temperature. Then the slides were rinsed with TBST and incubated with 100 µL of the anti-FITC antibody conjugated with horseradish peroxidase (HRP) during 30 min at room temperature in humidified chamber. Three 5 min washes with TBST were carried out and the samples were covered with a newly prepared solution of Blue Chromogen, which contains the substrate for HRP. The enzymatic reaction was carried out at room temperature for 10 minutes in a humidified chamber. The slides were successively washed with TBST, 1X TBS, and distilled water. Finally, the slides were dried by 30 min of incubation at 60°C.

It is worth of mention that the Dako DuoCISH kit allows the detection of signals from two different probes simultaneously on the same slide. However, since the PEN membranes were hybridized with only a single probe for HSA21, the kit reagents for detecting the antibody conjugated to alkaline phosphatase were not necessary.

### Imaging and Image Analysis

Stacks of images scanning the whole nucleus were acquired with an Olympus BX61 microscope fitted with a CCD camera, and coupled to a computer equipped with the Cell^M^ program. Then, we determined the frequency at which two chromosomes 21 were overlapping in at least two focal planes of the z-stack. Since we observed that there were cells with signals very close, but not juxtaposed, the absolute spatial separations between chromosomes were directly measured from their center of mass, and were normalized as a fraction of the nuclear diameter to account for natural variations in nuclear size. The frequency of the experimentally observed chromosome close positioning was statistically analyzed by contingency tables analysis, and applying Fisher's Exact test. T-Student Test (p≤0.05 significant) was used for comparing distances.

### Interphase Chromosome Microdissection

The microdissection was performed with an Olympus IX71 microscope equipped with the Laser P.A.L.M. system and the accompanying PalmRobo analysis software (Center Valley, USA). The procedure included two steps: laser microdissection and pressure catapulting of the samples. First, the system was calibrated to ensure that automatic laser functions were performed precisely at the required locations. Using 100X magnification the laser was set at 35 units of focus and 52 units of energy in five pulses/second to perform cutting at a normal speed. Once the laser cutting conditions were established, the pressure catapulting features were computationally established in the system (Cut+Delta = LPC). The cap holder was positioned into the line of the laser, directly above the objective and as close as possible to the sample (0.5–1 mm). Chromosome 21 territories, observed as blue precipitate in the nucleus, proximally located to one another were selected for microdissection. The computer program was used to draw a region of interest encompassing the two chromosomes on the live-image of the nucleus, which was used to guide laser cutting of the membrane. Chromosomes were collected into the cap of a microcentrifuge tube by pressure catapulting. Using the software function “go to checkpoint”, the slide was moved out of the light path and the cap was lowered towards the microscope’s objective lens to verify the presence of the specimen by direct observation. The material was then stored in individual tubes at −20°C until use in amplification.

### DNA Amplification

Whole genomic DNA amplification (WGA) was performed with the GenomePlex WGA4 kit (Sigma-Aldrich, USA) following the manufacturer’s instructions, which includes three main steps: lysis and fragmentation, library preparation and amplification. Briefly, 9 µL of water were added directly into the cap to dissolve the chromosomal DNA and the tube was immediately centrifuged (12000 rpm, 5 min) to transfer the solution to the bottom of the tube. Then, 1 µL of freshly prepared lysis solution containing proteinase K was added to the sample, mixed thoroughly, incubated for 1 h at 50°C, and heat inactivated by incubation at 99°C for exactly 4 min. This incubation step denatures double stranded DNA into single stranded DNA and facilitates non-enzymatic fragmentation to generate randomly fragmented DNA of overlapping short templates of 200 to 1,000 base pairs in length. Subsequently, the DNA fragments were efficiently primed to generate the Omniplex library, consisting of DNA fragments converted into PCR-amplifiable units that are flanked by universal adaptor sequences. The library was generated by adding 2 µL of library preparation buffer (containing degenerate adapters and stabilization solution) to the sample and incubating for 2 min at 95°C. Then, 1 µL of library preparation enzyme was added and the sample mixture was placed in a thermal cycler (Bio-Rad Laboratories, USA) to anneal universal adapters to the 5′ and 3′ ends of each DNA fragment by the following sequential incubations: 16°C for 20 min, 24°C for 20 min, 37°C for 20 min, and 75°C for 5 min. At this point, the PCR amplification procedure was performed by adding 7.5 µL of 10X amplification master mix (containing adapter specific primers), 5 µL of WGA DNA polymerase (Sigma-Aldrich, USA), and 48.5 µL of nuclease-free water directly to the tube. The reaction mix was returned to the thermal cycler and amplified by the following PCR conditions: 95°C for 3 min, followed by 25 cycles of 94°C for 30 s and 65°C for 5 min. The DNA concentration was spectrophotometrically determined (ND-1000; Nanodrop Technologies, USA), and the DNA quality was evaluated by electrophoresis through a 1.5% agarose gel. The PCR product produced the characteristic smear of fragments (range: 100–1000 bp).

A secondary amplification was performed with at least 10 ng of WGA-DNA (1 µL) using the GenomePlex DNA Reamplification kit (WGA3) (Sigma-Aldrich, USA), following the manufacturer’s instructions. The reaction components were removed by the PCR Clean-Up kit (Qiagen, Germany). The amplified DNA from dissected chromosomes and total genomic DNA from the same cells were stored at −20°C.

### Allele-specific PCR

To identify chromosomes that localize in close proximity, we performed PCR with primers specific for microsatellite markers mapping on chromosome 21.

We selected the D21S11, D21S1270, D21S1411 and D21S1435 microsatellites markers (Table 1), based on the fact that they are commonly used in clinical practice to determine the parent-of-origin of the supernumerary chromosome in DS patients. The PCR were performed on total genomic DNA extracted directly from cell cultures and DNA from the microdissected chromosomes as previously described [11]. Briefly, 100 ng of DNA were mixed with 5 µL of Hot Start Taq PCR Master Mix (Qiagen, Germany), specific primers, and double-distilled water in a 10 µL final volume. The concentration of the primers varied for each reaction, as follows: 0.2 µM for D21S1435; 0.24 µM for D21S11; 0.8 µM for D21S1270; and 0.8 µM for D21S1411. The PCR conditions included an initial enzyme activation incubation step of 94°C for 15 min, and five preliminary cycles consisting of a denaturation step at 94°C for 30 s, an annealing step at 60°C for 30 s and an extension step at 72°C for 30 s, which was followed by 30 amplification cycles of 94°C for 30 s, 57°C for 30 s and 72°C for 30 s, and a final extension at 72°C for 20 min. The performance of the reaction was initially assessed by electrophoresis on agarose gel (1.5%) and visualization under the UV lamp. The fragments had the expected size (160–200 pb), however precise length and concentration of PCR products was determined by capillary electrophoresis in a Bioanalyzer 2100 using the DNA1000 LabChip kit and accompanying 2100 Expert software (Agilent Technologies, USA), following the manufacturer’s instructions.

**Table 1:** Primers, addressed to microsatellites mapping to chromosome 21, used in the allele specific PCR of this study.

Marker	Primer 5′→3′	Product size (bp)
D21S11	TTTCTCAGTCTCCATAAATATGTG	225–280
	GATGTTGTATTAGTCAATGTTCTC	
D21S1270	CTATCCCACTGTATTATTCAGGGC	285–340
	TGAGTCTCCAGGTTGCAGGTGACA	
D21S1411	ATAGGTAGATACATAAATATGATGA	256–340
	TATTAATGTGTGTCCTTCCAGGC	
D21S1435	CCCTCTCAATTGTTTGTCTACC	160–200
	ACAAAAGGAAAGCAAGAGATTTCA	

Specific PCR with unamplified total genomic DNA extracted from diploid and Down syndrome cell cultures was performed to establish the allele status without potential bias due to the amplification steps. Allele-size of microdissected chromosomes was compared with that obtained from genomic DNA. Parents genotype was not examined because the purpose of the study was to determine whether the same chromosomes localize juxtaposed, rather than the parental origin of these chromosomes.

**Figure 1:**
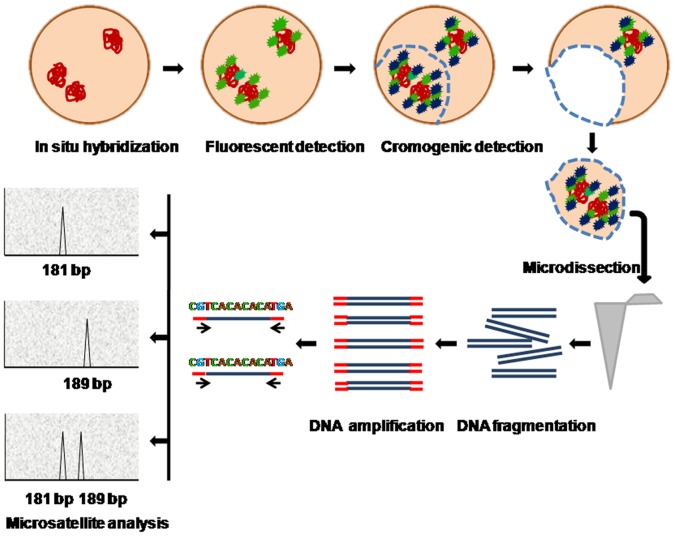
Schematic of the FISH-CISH procedure combined with microdissection and microsatellite allele genotyping. *In situ* hybridization is performed on cells spread onto PEN membranes. The detection step is performed first with fluorescent antibodies followed by quality inspection under the fluorescence microscope. Then, a chromogenic detection step and the microdissection of selected chromosome pairs are performed under the bright-field microscope. DNA is amplified by whole genome amplification and chromosome identification is performed by allele-specific PCR.

The experimental scheme is depicted in figure 1.

## Results and Discussion

### Localization Pattern of Chromosomes 21 in Down Syndrome Nuclei

**Figure 2:**
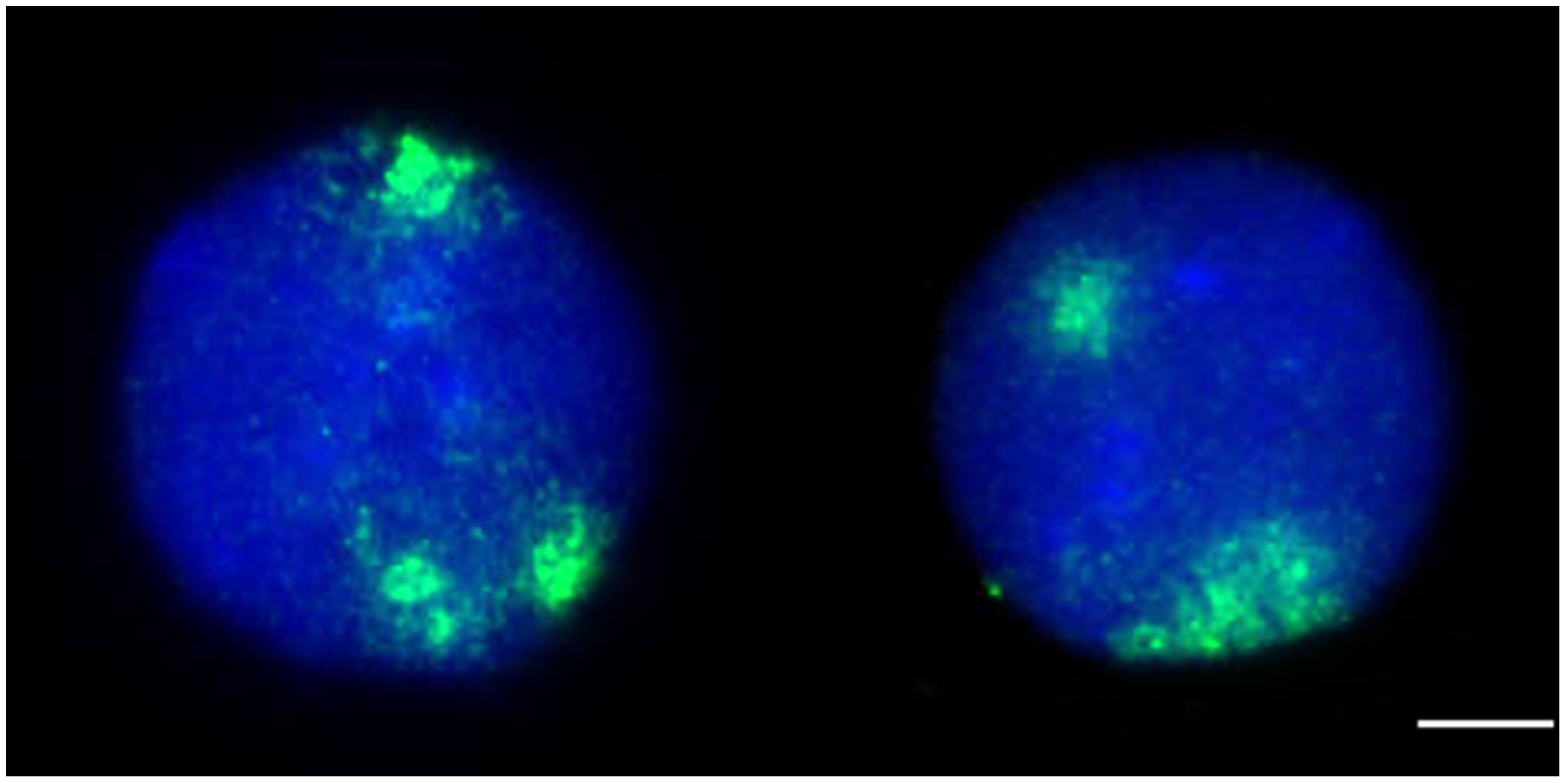
Chromosome 21 proximal positioning. FISH analysis with whole painting probes for chromosome 21 (green) showing the most frequent distribution in trisomic cells. Counterstaining with DAPI (blue), scale bar = 4 µm.

Chromosomes are not randomly distributed in the nuclear space [2]. Size and gene density are two factors related to chromosome positioning [12], [13]. Not only have these structural parameters been related to nonrandom distribution of chromosomes within the interphase nucleus, but functional features, such as gene transcription and DNA replication and repair have been shown to be spatially organized within the interphase nucleus [14]. We observed a distinctive positioning pattern of the three copies of chromosome 21 in the interphase nucleus of cells (N = 58) from human with DS, in which two chromosomes localize in close proximity to each other, and distant from the remaining chromosome in 55% of cell-nuclei (Figure 2). The analysis also showed that only 17% of trisomic cells had three distant signals, while 28% exhibited one unique cluster including all three CT 21. Homologous association was detected in 44% of nuclei from diploid cells (n = 34). Fisher test demonstrated that the tendency of CT 21 association is statistically different (p = 0.00) in trisomic respect to diploid cells. To further analyze chromosomes 21 relative positioning, we measured the distances between CT 21. We found that the two chromosomes located in close proximity were separated by an average distance equivalent to 30% of the nucleus diameter (ND), while mean distance to the third chromosome was 53% ND. Statistics demonstrated that in nuclei from trisomic cells the two CT 21 arranged close to one another are at a distance significantly lower than the interchomosome distance (47% ND) found in diploid cells (p = 0.00). Whereas the third CT 21 is located at a distance which is similar to the interchomosome distance found in diploid cells (p = 0.10). In previous studies we established that two chromosomes separated by less than 30% of the nuclear diameter are forming a spatial pair [15]. Nagele and coworkers investigated the pattern of distribution of chromosomes in prometaphase rosettes. By measuring the angular separation, they found that in triploid cells two homologues chromosomes were closely juxtaposed (angular separation = 22.6±11.1°), while the other chromosome remained in the opposite side of the rosette (angular separation = 162.10±18.8°) [16]. Our results that two homologues 21 are preferentially positioned in close proximity within the interphase nucleus of trisomic cells are in agreement with those obtained by Nagele and coworkers for triploid cells. The spatial association of the maternal and paternal chromosome region 15q11-q13 has been observed in cells from patients with Prader-Willi and Angelman syndrome, which is believed to facilitate the establishment and maintenance of gene imprinting [17]. However, it has been recently suggested that such association would be a side effect of the association of chromosomes 15, which harbor NORs, in a single nucleolus [18]. A similar mechanism may underlie the observed proximal positioning of two chromosomes 21 in trisomic cells, but the question of whether this pairing in the interphase nucleus is chromosome-specific remains.

### Combined FISH-CISH Approach to Genotype Interphase Chromosomes

Proximal positioning of two chromosomes 21 in trisomic cells can also be explained by the functional association of homologous chromosomes around the same nucleolus. In other words, coordinated transcription of ribosomal DNA may be determining such distribution. We asked, however, whether the same chromosomes 21 form this proximal pair in all cells. To answer this question, we designed the approach presented here, which consists of FISH and CISH sequential techniques followed by microdissection and alellotyping of juxtaposed chromosomes. The procedure includes many steps (Figure 1); therefore the slides should be handled carefully, in particular during post-hybridization washing steps. Using soft instruments to physically transfer slides from one recipient to another or gently pouring solutions is strongly recommended, to avoid damaging the PEN membrane. It is also important to maintain the slides humid during the entire procedure to avoid generation of background signals. However, the slides must be dried completely at the end of the process and no mounting medium should be used, because any liquid on the membrane impairs the laser microdissection and catapulting processes.

Hybridizations were performed with probes labeled with Digoxigenin-11-dUTP and Biotin-16-dUTP, and the fluorescent detection was assayed with an antibody against Digoxigenin conjugated with FITC and avidin, respectively. The anti-Digoxigenin-FITC was selected for the initial part of the procedure because it rendered less unspecific signal (background) than the biotin-avidin detection method. FISH has been successfully used in a wealth of diverse applications, such as prenatal and preimplantation diagnosis of aneuploidy, and monitoring the evolution of patient with oncological diseases carrying chromosome abnormalities [19], [20]. CISH, on the other hand, has an advantage over the FISH procedure because it uses bright-field microscopy; therefore it is broadly used among pathologists [21]. We attempted to use each method separately, and we found that the fluorescent detection method alone is not suitable for the microdissection step because requires mounting the preparation, while CISH alone does not yield strong signal on preparations made on membranes. It is well known that the signal intensity could be improved by increasing the number of antibody layers irrespectively of the use of fluorochrome-tagged antibodies. However, the advantage of combining CISH with FISH is to have a robust and quick quality control assay to ensure the hybridization was specific at the beginning of procedure, and not strictly to improve CISH signal.

**Figure 3:**
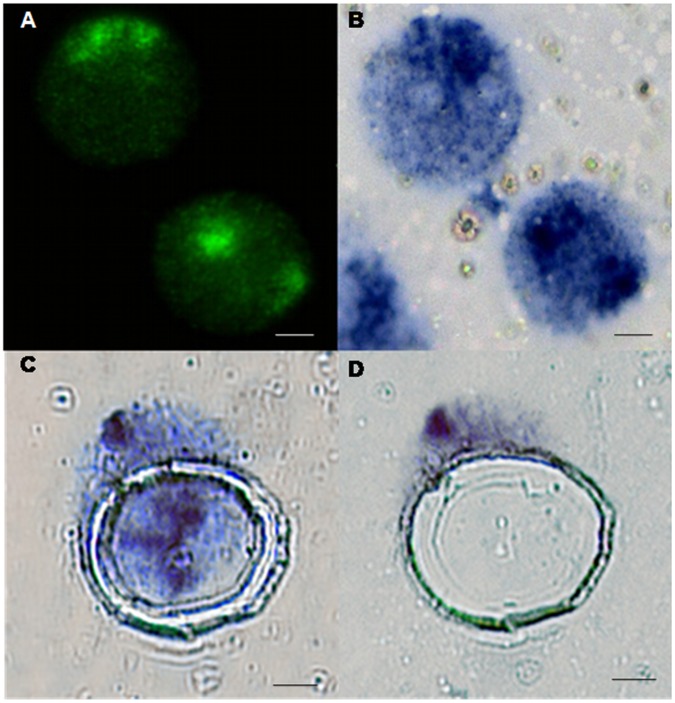
Hybridization with HSA21 DNA probes on cells spread onto PEN membranes. (A) FISH signal (green) in interphase nuclei. Note the two juxtaposed chromosomes 21 are distant from a third chromosome in the nucleus of cell from human with DS. (B) Subsequent CISH (blue) on the same preparation. Chromosome pairs are visible as blue precipitate under the bright-field microscope. (C) A region of interest is computationally drawn and the membrane is cut with the laser microbeam (green). (D)The piece of membrane harboring the chromosomes is recovered by pressure catapulting (blank white space).

The combined fluorescent-chromogenic method that we implemented resulted in clearly distinguishable interphase chromosomes (Figure 3A). Visualization of the fluorescent signal during the procedure, allowed us to control the hybridization quality and search for suitable nuclei. The blue precipitate in the nucleus (a product of the subsequent chromogenic detection) permitted the laser microdissection of interphase chromosomes (Figure3B).A total of 14 proximally positioned chromosome pairs were dissected from three different experiments, of which 60% were juxtaposed chromosomes (Figure 3C, D).

### WGA and Molecular Analysis

**Figure 4:**
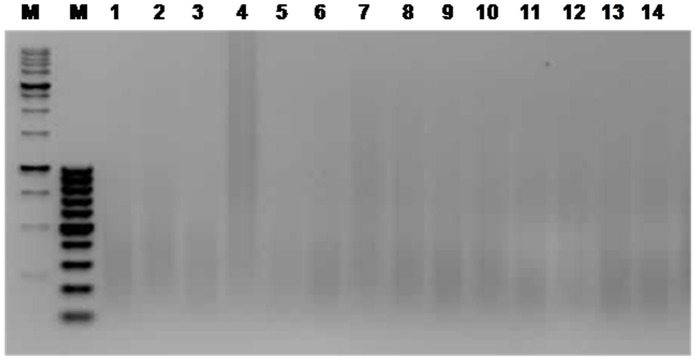
WGA efficiently amplified all 14 samples of microdissected chromosome 21 pairs. Lanes: M, molecular weight markers from 25–500 bp (far left) and 100–1000 bp (second to left); 1–14, chromosome samples.

The dissected material was subjected to WGA with optimized conditions for 10 ng DNA, which yielded a smear of fragments ranging ≈100 to ≈1000 bp in size. Following this procedure we obtained approximately 1 ug of DNA from each microdissection experiment (910±115 ng). However, the efficiency of the WGA procedure varied among samples, most likely due to quality differences of the original material (Figure 4).

### Identification of Chromosome 21 Alleles

**Figure 5:**
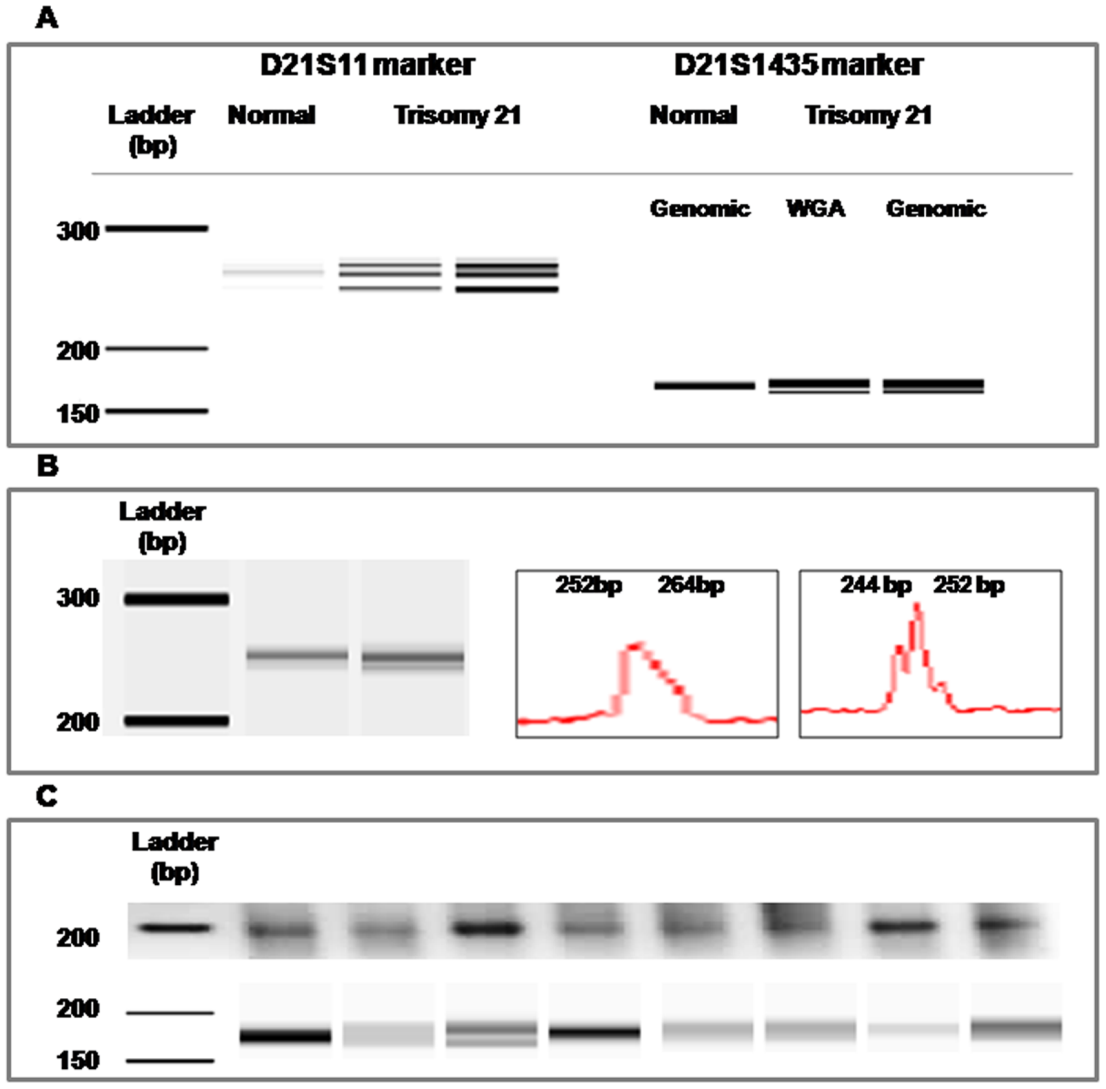
WGA and allele-specific PCR analysis. (A) Allele-specific PCR with primers for the D21S11 and D21S1435 markers on WGA-DNA and genomic-DNA from trisomic cells yielded the same allele profiles. (B) Capillary electrophoresis in a Bioanalyzer of the PCR products for D21S11revealed the presence of two different alleles in two microdissected chromosome pairs. (C) PCRs with primers for the D21S1435 marker produced fragments of ∼200 bp in a 1.5% agarose gel (upper panel). Capillary electrophoresis in a Bioanalyzer revealed the presence of two alleles within this band (181 bp and 189 bp) in a limited number of microdissected chromosome pairs (lower panel).

**Figure 6:**
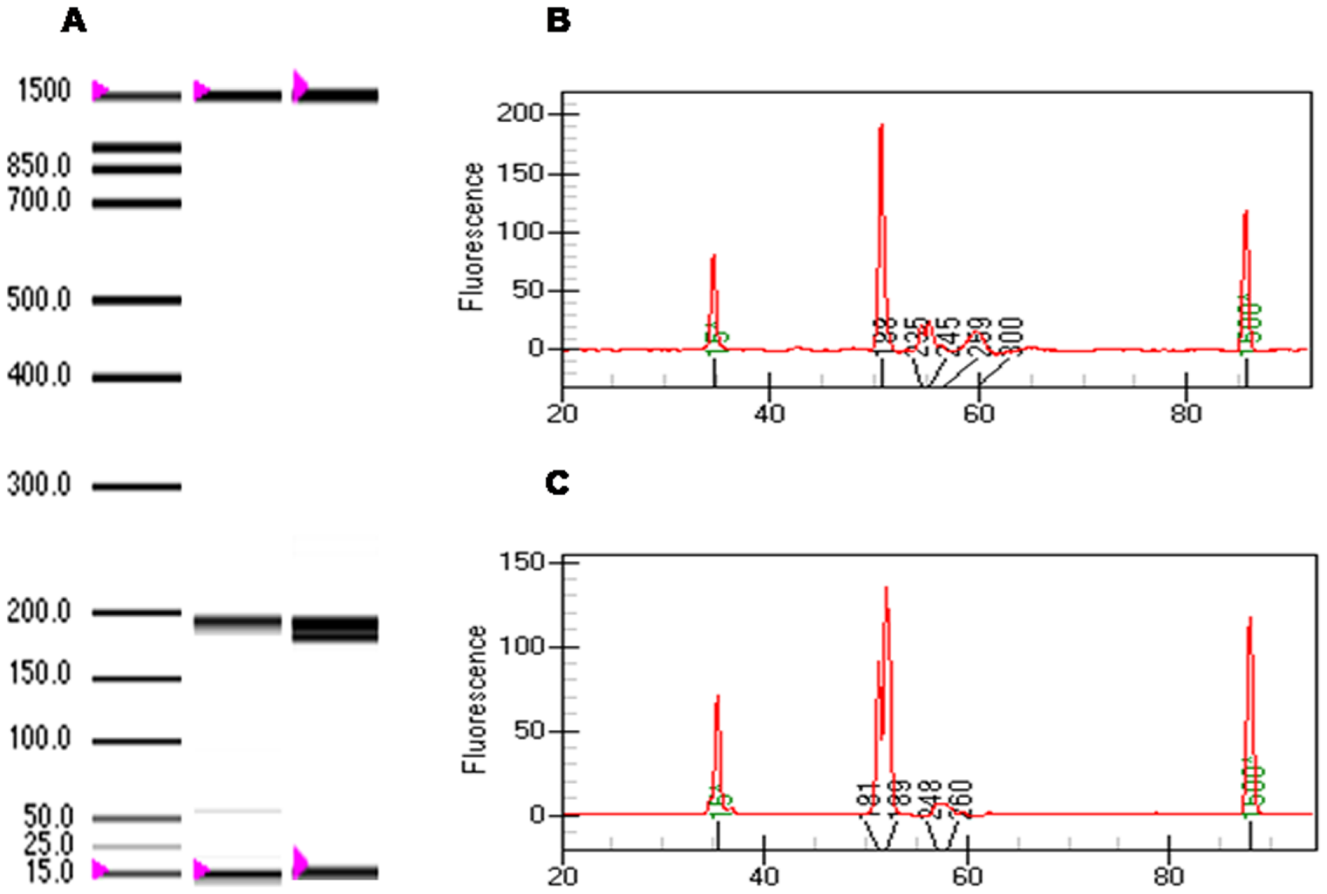
Genotype profile for the D21S1435 marker. (A) Left lane correspond to the internal DNA-size marker. The first and the last band on each lane correspond to DNA-size marker of 15 bp and 1500 bp, respectively. Middle lane, single band (189 bp) obtained from microdissected juxtaposed chromosomes having the same microsatellite allele. Right lane, two bands indicate the presence of two alleles in the microdissected material. (B) Electrophoregram showing the single peak of 189 bp from lane 1 in (A). (C) Electrophoregram showing the two alleles (181 and 189 bp) from lane 2 in (A); this profile corresponds to the cell shown in Figure 3C.

In order to determine the identity of the chromosomes 21 forming the pair in the interphase nucleus, we performed PCR specific for microsatellites mapping on this chromosome (see Material and Methods). First, we performed the alellotyping with primers for all microsatellites markers on genomic DNA from the normal cell line (disomic) and the trisomic cell line, and found that the trisomic cell line is heterozygous for D21S11 (3 alleles) and D21S1435 (2 alleles). Whereas the normal cell line was homozygous for both markers. Similar results were obtained with genomic-DNA subjected to WGA, indicating that this last procedure does not induce allele drop-out (Figure 5A). Electrophoresis of the PCR products for D21S1270 and D21S1411 gave single bands (non informative). Then, we performed the same reactions with primers for D21S11 and D21S1435 on WGA-DNA from 14 microdissected chromosome pairs. Amplification was obtained from four out of the 14 samples for the D21S11 marker, and capillary electrophoresis of the PCR products showed that in two cells the microdissected chromosomes harbored different alleles (Figure 5B). The remaining two chromosome pairs gave broad bands that could not be resolve. PCRs with primers for the D21S1435 marker were more efficient, 10 samples gave products. Electrophoresis on agarose gel showed that the products of the PCR with primers for this marker produced a single band of ∼200 bp (Figure 5C). However, Bioanalyzer capillary electrophoresis revealed that five microdissected pairs were composed by chromosomes harboring the same allele, including one sample which gave a faint band in the zone of the 189 bp (Figure 5C, lane 7). This procedure also showed that this band was composed of two fragments (181 bp and 189 bp) in one sample (Figure 5C). Furthermore, we obtained broad bands that seemed to include both alleles in only one additional sample (Figure 5C, lane 2), and a single allele in three microdissected chromosome pairs (Figure 5C, lanes 5, 6, 8). Based on the results from the five microdissected chromosome pairs which yielded well defined bands for this marker, we determined that four pairs were composed of chromosomes carrying the same allele, and the remaining pair was formed by chromosomes with different alleles, similar to the profile obtained with genomic DNA (Figure 6).

Chromosome microdissection provides a direct approach for isolating DNA from entire chromosomes or any recognizable region within. Developed over 20 years ago, this procedure has emerged as a powerful tool of genomic research, facilitating physical map construction and generation of FISH probes. Its use to date, however, has relied exclusively on metaphase spreads and pools of chromosomes from several cells as a source of chromosomes [22]. To the best of our knowledge, our current study represents the first report of interphase chromosome microdissection.

The DNA yields after microdissection were not sufficient for direct microsatellites analysis, therefore two rounds of amplification were required. The risk of DNA contamination is an important problem for single-cell PCR procedures [23]. However, under the conditions described here, the DNA profiles obtained with our method permitted us to rule out the presence of DNA contamination. Furthermore, the product of WGA control reactions performed without DNA and a piece of PEN membrane without any cell material yielded the expected negative results.

The genetic analysis of the chromosome pairs subjected to microdissection revealed that the juxtaposed chromosomes have different alleles of the microsatellite marker D21S1435 (Figure 6). Furthermore, and despite the limited number of samples, the results obtained with the microsatellite marker D21S11 support the notion that the chromosomes proximally positioned are not always the same. However, the data obtained for D21S1435 suggest that there may be tendency of the same chromosomes to be positioned close to each other in the interphase nucleus of trisomic cells. Ma et al. have recently reported a method for the determination of haplotypes through chromosome microdissection, but again by using metaphase spreads [24]. On the contrary, the protocol described here was designed for the microdissection of interphase chromosomes in single cells, representing a clear improvement for genome analysis.
